# Cognitive control and adaptive responding in patients with Attention Deficit Hyperactivity (ADHD) and Autism Spectrum Disorder (ASD)

**DOI:** 10.1192/j.eurpsy.2025.2052

**Published:** 2025-08-26

**Authors:** B. Kakuszi, I. Bitter, P. Czobor

**Affiliations:** 1 Psychiatry and Psychotherapy, Semmelweis University, Budapest, Hungary

## Abstract

**Introduction:**

Deficits of cognitive control and performance monitoring play a critical role in the psychopathological manifestations of ADHD and ASD. However, relatively few studies have used a transdiagnostic approach to examine deficits in cognitive control and performance monitoring deficits across diagnostic boundaries.

**Objectives:**

Using a transdiagnostic approach, we examined post-error slowing (PES), a principal measure of cognitive control and adaptive behavior, in subjects with ADHD and ASD compared to typically developing (TD) subjects. We also investigated the signal detection ability in the three study groups using the d-prime index, which characterizes the observer’s ability to select the right stimuli while avoiding the wrong ones based on the commission and omission errors.

**Methods:**

Participants included adults (18-65 years) with the DSM-IV diagnosis of ADHD (n=22) or ASD (n=24), as well as TD subjects (n=25). We used pictures from the International Affective Picture System as stimuli, displayed in random sequence. Stimuli were shown centrally every 1400 msec for 800 msec. A total of 243 stimuli were shown in two blocks (negative, positive & neutral pictures with equal probability). Subjects were asked to push a button as soon as possible upon appearance of the stimulus pictures (Go trials); they were, however, asked not to respond if a picture was repeated (NoGo trials). Generalized Linear Model (GENMOD) analysis was used to test post-error slowing (decrease of reaction time after an error) and the d-prime index, applied as dependent variables in the analyses. Study group (ADHD, ASD, TD) was used as independent variable.

**Results:**

The analysis indicated a significant (p<0.05) overall group difference in PES among the three study groups. Post-hoc analyses showed that as compared to TD subjects, patients with ADHD manifested a markedly increased PES (˜70msec, p<0.05), while subjects with ASD showed no significant change. Additionally, we found a significantly reduced value for the d-prime index in both the ADHD and ASD groups as compared to TD subjects, with no difference between the ADHD and ASD group.

**Conclusions:**

While the reduction of signal detection ability was similar in the ADHD and ASD groups, subjects with ADHD and ASD showed a distinctive profile of post-error adjustment in a behavioral response inhibition task. While patients with ADHD show decreased inhibition and fail to make the adjustment that TD subjects make (as indicated by the decreased PES in ADHD), adults with ASD showed intact behavioral reactions after errors (i.e., with post-error slowing reactions similar to that observed in TD subjects).

**Funding:**

**Hungarian Brain Research program,#NAP2022-I-4/2022**

**Disclosure of Interest:**

None Declared

